# Testing Polynomial (Non-Linear) Effects of Neurological and Physical Disability on Sexual Well-Being in Multiple Sclerosis

**DOI:** 10.3390/brainsci14121177

**Published:** 2024-11-25

**Authors:** Rafał Gerymski, Maria Latusek-Mierzwa

**Affiliations:** 1 Department of Health Psychology and Quality of Life, Institute of Psychology, Opole University, 45-040 Opole, Poland; 2 Doctoral School of the Opole University, 45-040 Opole, Poland; maria.latusek-mierzwa@uni.opole.pl

**Keywords:** sexual well-being, sexual dysfunctions, disability, multiple sclerosis

## Abstract

Background: Multiple sclerosis (MS) is among the most prevalent chronic autoimmune disorders affecting the central nervous system. In Poland, the MS incidence rate is 6.3 per 100,000 patients. The results of studies indicate that people suffering from MS are less involved in sexual life in both the physical and emotional aspects, they assess that sex is less important to them than to healthy people, and their sexual motivation is limited, especially its physical dimension. Methods: A total of 121 people participated in the study. It used four questionnaires: authors’ survey on sociodemographic and biomedical data, Short Sexual Well-Being Scale, Guy’s Neurological Disability Scale, and Expanded Disability Status Scale. Results: A positive and moderate relationship was found between neurological disability and physical disability. Also, the correlation between neurological disability and the number of sexual dysfunctions was significant. Problems in the neurological sphere were also negatively associated with sexual well-being. Physical disability was not significantly associated with the level of sexual well-being or the number of declared sexual dysfunctions. The number of dysfunctions was moderately associated with the levels of sexual well-being. The polynomial regression analyses did not find any non-linear relationships between the severity of disability and sexual well-being. Conclusions: This study suggests that the occurrence of sexual dysfunctions can be an important subject in MS patients’ sexual well-being. It underlines the fact that the subject of one’s sexuality cannot be omitted while providing medical and psychological support for individuals with MS.

## 1. Introduction

Multiple sclerosis (MS) is a chronic autoimmune disease of the central nervous system. Over 2.2 million people suffer from it worldwide [1]. In Poland, the MS incidence rate is 6.3 per 100,000 patients [2]. It is most often diagnosed between the ages of 20 and 40, and the incidence of MS is twice as frequent in women than men [3]. It is an incurable disease which manifests itself in fully or partially reversible episodes of neurological and physical disability. Typical symptoms occurring during episodes include monocular vision loss caused by inflammation of the optic nerve, double vision, limb weakness or loss of sensation, problems with concentration, and difficulty in moving. About 10–20 years after the diagnosis, the disease becomes progressive, which ultimately leads to impairment of cognitive functions and mobility. Data show that 15% of people with MS struggle with progressive disease from the beginning of its onset and diagnosis [4].

The exact cause of MS is unknown, but it is known that genetic and environmental factors play an important role [1]. Progressive physical and neurological disability in MS has significant consequences for the functioning of people affected by this disease. Over the course of MS, patients show a decrease in their level of activity during the day and mood swings. In turn, limitations related to fatigue, physical weakness, and more frequent problems with maintaining balance and coordination appear, and the ability to maintain concentration decreases. In addition, there is a change in temperature sensitivity, muscle stiffness, motor impairment, and sleep problems. All this is associated with a decrease in the self-esteem and well-being of people suffering from MS, limited interpersonal contacts, and increasing difficulties in entering into new relationships. People with MS are therefore at high risk of depression and various psychological problems [5]. Due to persistent symptoms leading to neurological and physical disability, difficulties in accepting the disease may occur, which may additionally reduce the quality of life of people with MS [6,7,8].

The course of the disease itself may also be differentiated by gender. Women show more inflammatory symptoms in episodes of disease relapse, but only until menopause. After this time, the gender differences in this regard disappear. On the other hand, men are more susceptible to the neurodegenerative symptoms of MS [9]. Women more often complain about fatigue, pain, and sensory disorders, while men more often declare a sense of weakness. There are also differences in the psychological problems experienced. Women more often feel sad, and men show a greater lack of acceptance of the disease. In the group of people diagnosed with MS, there are also more suicide attempts compared to the general population, which may be the result of an increased level of depression in this group [10]. In summary, MS is a disease that significantly affects human life, impairing physical and neurological functioning and leading to disability. Unfortunately, little attention is paid to the sexual life of patients with multiple sclerosis.

The neurological symptoms of MS can affect the physical and mental functioning of patients, as well as the sexual sphere [11]. This can be associated with the occurrence of sexual dysfunctions, which can be diagnosed in about 50% to even 90% of patients suffering from MS [12,13,14,15]. Sexual dysfunctions in those individuals can be a result of sensorimotor impairment, disorders in the functioning of the central nervous system, and reduced cognitive functions [16]. Among men with MS, erectile dysfunctions, orgasmic dysfunctions, and reduced libido are the most commonly diagnosed sexual dysfunctions [17]. In the group of women with MS, the most frequently declared problems in the sexual sphere are anorgasmia, reduced libido, lubrication problems, pain, muscle spasticity, and worries about the sexual satisfaction of their partner [15,18]. Those who complain of a higher level of physical disability assess their sexual functioning worse, and the level of satisfaction with their sexual life is lower compared to healthy women [19].

The results of the comparison of groups of healthy people and people with MS indicate that people suffering from MS are less involved in sexual life in both the physical and emotional aspects, they assess that sex is less important to them than to healthy people, and their sexual motivation is limited, especially its physical dimension [20]. On the other hand, despite the high prevalence of sexual dysfunctions, studies indicate that over 50% of women assess that their sexual life has not changed after the diagnosis of MS [21]. According to some studies, the duration of the disease is not a factor that would be related to the assessment of sexual life and sexual dysfunctions [11,12,22]. Other studies suggest that the occurrence of chronic disease is associated with a lower level of sexual well-being, which can be associated with both the occurrence of sexual dysfunctions and acceptance of illness. Due to a lack of acceptance of illness, patients may struggle with psychological problems such as higher levels of stress or lower levels of self-esteem. The occurrence of this kind of issue may contribute to a decreased level of sexual well-being and increase the levels of dysfunctions [23,24,25].

Sexual well-being refers to the emotional, sexual, and cognitive fulfilment one experiences regarding their sexuality. It differs from sexual satisfaction, which pertains solely to the physical aspect of sexuality. This intricate concept encompasses various elements, including sexual health, physical enjoyment, mental contentment, and sexual anxiety [23,24,25,26]. It is not clear which factors are the most important in the sexual well-being of MS patients. Furthermore, some data suggest that despite many difficulties related to the course of multiple sclerosis, this chronic disease does not have to negatively affect the sexual sphere of life of those affected by it [21]. This can be explained by two things: the occurrence of some protective factors or the fact that the relationship between neurological and physical impairment and well-being is non-linear. To date, no scientific study has tested the non-linear relationship between various aspects of the functioning of patients with MS and their sexual well-being. Therefore, this study analyzed the sexual well-being of MS patients and its correlates, such as patients’ age, disease duration, levels of neurological and physical disability, and the number of sexual dysfunctions.

## 2. Materials and Methods

### 2.1. Participants and Procedure

This paper discusses the findings from an online cross-sectional study. The data included in this study were gathered during the period from 2022 to 2023. Participants were recruited through social media platforms and foundations for people with multiple sclerosis. This recruitment method was used to ensure the respondents’ sense of anonymity, which is particularly important in the case of research on sexuality. A total of 121 people participated in the study. The criteria for inclusion were specified as follows: (1) being an adult (18 years and older); (2) not having cognitive deficits; (3) having the ability to make independent decisions; (4) being a native Polish speaker; (5) having a diagnosis of multiple sclerosis. The exclusion criterion was an episode of hospitalization in the last 6 months. The above information was verified based on the respondents’ statements.

All participants were notified about the confidentiality of the results obtained and about their right to withdraw from the study at any time. The study was designed and conducted following the guidelines of the Bioethics Committee at the Institute of Medical Sciences of the University of Opole (directed by the Act of 5 December 1996, on the professions of physician and dentist, issued by the Sejm of the Republic of Poland) and by the ethical principles of the Helsinki Declaration. The presented study is part of an ongoing project focused on sexual well-being in the Polish community carried out by the first author (Opole University’s Research Ethics Committee approval number: KEBN 1/2021; approved on 20 January 2021).

### 2.2. Materials

The study used four questionnaires. All sociodemographic and MS-related data were measured using a survey by the authors. It consisted of closed and open-field questions about participants’ health and status, such as gender, age, disease duration, form of multiple sclerosis (primary progressive, secondary progressive, progressive–relapsing, relapsing–remitting, undefined), education, civil status, sexual activity (“Are you sexually active?”) and sexual dysfunctions (lack or loss of sexual desire, lack of sexual pleasure, lack or delay of orgasm, erectile dysfunction, vaginal dryness, premature orgasm, vaginismus, dyspareunia, vulvodynia, pain during intercourse, pain in intimate areas, other and the question “Do you believe that MS affects your sexual life?”).

Sexual well-being was assessed using the Short Sexual Well-Being Scale (SSWBS) [26]. It is a 5-item scale, where: 0—“I strongly disagree”; 7—“I strongly agree”. A higher SSWBS score means a higher level of subjective sexual well-being. In the presented study, the scale had good reliability (Cronbach’s alpha = 0.87).

The severity of neurological disability was measured using Guy’s Neurological Disability Scale (GNDS) [27]. The questionnaire includes 72 questions using a dichotomous nominal scale, with responses as 1 for “Yes” and 0 for “No”. In this study, the summary score was calculated. A high GNDS score indicates a high level of perceived neurological disability. In the current study, GNDS showed good reliability (Cronbach’s alpha = 0.71).

Physical disability was operationalized with the Expanded Disability Status Scale (EDSS) [28]. The EDSS consists of 20 levels of disability, with each degree scored with every half point. A higher score on the scale indicates a greater level of disability, where: 1—“No disability”; 5—“Disability severe enough to impair full daily activities”; 10—“Death due to MS”. Because of the characteristics of the EDSS, it is not possible to compute standard reliability coefficients like Cronbach’s alpha.

### 2.3. Statistical Analysis

All results were analyzed using JAMOVI 2.6.13 statistical software [29]. Differences between the studied groups were verified with the *t*-test analysis. The relationships between the studied variables were measured with Pearson’s r correlation, linear regression, and polynomial (non-linear) regression. A significance level of α = 0.05 was established as the cutoff for statistical significance in all analyses.

## 3. Results

### 3.1. Sociodemographic Characteristics of the Studied Sample

Initially, 121 people took part in the study. Based on the inclusion/exclusion criteria, 113 people were qualified for the study. The study sample consisted of 53 women and 60 men aged from 18 to 65 (M = 45.59; SD = 11.76). The majority consisted of people with secondary education (46.02%), who were married (65.49%). The average duration of the disease was 12.03 years (SD = 7.15). Additionally, 94.69% of the respondents were sexually active (based on the subjective evaluation of the study participants by their definition of “sexual activity”), and 93.81% believed that their disease affected their sexual sphere in some way. Most respondents struggled with the relapsing–remitting form of multiple sclerosis (91.15%) and declared an average of 1.38 sexual dysfunctions (SD = 1.83). Unfortunately, due to the numbers in the described subgroups, it was not possible to examine intergroup differences using variables other than gender.

### 3.2. Group Differences

First, the differences between the study participants were verified. For this purpose, the *t*-test for independent samples was used. It was shown that men were significantly older than women (based on Cohen’s d effect size coefficient, it was a small difference). Moreover, women obtained significantly higher scores in the severity of physical disability (small difference). The remaining differences in MS’s duration, neurological disability, number of declared sexual dysfunctions, and sexual well-being were not statistically significant. On this basis, it should be assumed that the tested sample can be treated as homogeneous in subsequent analyses. For more detailed results, see Table 1.

### 3.3. Relationships Between Variables

The relationships between the studied variables were verified using Spearman’s rho correlation due to the ordinal nature of one of the variables—the number of sexual dysfunctions. A positive and strong relationship was found between the age of the subjects and the duration of the disease. Moreover, age was positively and weakly associated with the severity of neurological disability—the higher the age, the worse the functioning in the neurological sphere. The duration of multiple sclerosis was not significantly associated with the severity of disability or functioning in the sexual sphere. However, a positive and moderate relationship was found between neurological disability and physical disability. Also, the correlation between neurological disability and the number of sexual dysfunctions was significant. Additionally, problems in the neurological sphere were also negatively associated with the level of sexual well-being. Physical disability was not significantly associated with the level of sexual well-being or the number of declared sexual dysfunctions. On the other hand, the number of dysfunctions was negatively and moderately associated with the levels of sexual well-being—the higher the number of declared sexual dysfunctions, the worse the well-being in this sphere. Other relationships were non-significant. Detailed data are presented in Table 2.

Lastly, it was decided to test which of the tested variables would act as significant predictors of the subjective well-being in the studied sample of patients with MS. For that purpose, the linear regression analysis was used. The model was built based on the significant correlations presented in Table 2. Linear regression showed that only the number of sexual dysfunctions acted as a significant predictor of the tested dependent variable. Cohen’s *f*^2^ values indicate that the % of sexual well-being variance explained by the sexual dysfunctions can be interpreted as a medium effect. For more information, see Table 3.

Due to the strong empirical and theoretical background on the relationship between biomedical variables and well-being in individuals with MS (see Introduction), it was decided to test non-linear effects with the use of polynomial regression. For that purpose, two functions were used: quadratic function (which tests relationships that could be represented by the letter “U”) and cubic function (relationships which can be visualized by the letter “N”). The analysis showed that all tested relationships were characterized by marginally low effect sizes. Therefore, it can be concluded that in the studied sample there are no non-linear relationships between the studied variables (see Table 3).

## 4. Discussion

The results of this study might suggest that the diagnosis of MS is associated with various types of effects on functioning, including sexual functioning. Due to the occurrence of motor disability in the image of the disease, there are gender differences in the perception of the severity of this symptom. The findings of the present study show that women achieved higher scores on the physical disability scale. Research by Zhao et al. [10] found that, in a large group of people with MS, women reported higher levels of pain, fatigue, and sensory disturbances than men. Fatigue was also a symptom in other studies conducted in groups of people with MS [30]. These symptoms may be related to the severity of physical disability. Women may perceive their physical disability as higher compared to men, the course of the disease can be more severe in the female group, and this difference between the sexes evens out only after menopause in women [9].

In our study, the duration of the disease was significantly related to the age of the people studied in this study. This is a logical consequence of the fact that MS is a disease that progresses with age, most often diagnosed between the ages of 20 and 40. The level of neurological disability was also significantly (but weakly) related to the age of the people studied. A study conducted on a group of people with MS confirms this tendency, where over time the people studied assessed their functioning worse and the symptoms of neurological disability more frequently occurred [5]. 

In the present study, there are no gender differences in the occurrence of neurological disability, which is in line with a study by Magyari and Koch-Henriksen [9]. However, the severity of neurological disability significantly correlated with the level of physical disability. This is consistent with the symptoms that occur in MS. People suffering from this disease may exhibit symptoms of physical disability, as degenerative changes in the nervous system lead to decreased sensation, movement difficulties, and problems with maintaining balance [4]. Therefore, the correlation between these types of disabilities can be considered a characteristic feature of MS and it is not surprising that it also occurred in our study.

The results obtained in this study indicate a significant correlation between neurological disability and the number of declared sexual dysfunctions. The subjects who were characterized by a higher level of neurological disability indicated the occurrence of more sexual dysfunctions. Neurological disability is one of the symptoms of MS, and its occurrence can cause the occurrence of sexual dysfunctions. Numerous studies have confirmed the occurrence of sexual dysfunctions in patients with MS [14,17,18,31]. Symptoms of neurological dysfunction are associated with the occurrence of erectile and orgasmic disorders in a group of women and men [11], which is consistent with the results of this study. 

Moreover, this study showed that sexual well-being was significantly correlated with neurological disability. The higher the level of neurological disability, the lower the level of sexual well-being. Similar results were obtained in the studies by Alehashemi et al. [32] and Kaplan and colleagues [33], where the level of disability correlated with sexual satisfaction. Neurological disability, fatigue, and difficulty moving, which are symptoms of MS, may contribute to the lower level of sexual satisfaction in people struggling with this disease. However, the performed regression analysis showed that the levels of sexual dysfunctions are a more important predictor of sexual well-being than neurological disability. A greater number of sexual dysfunctions is associated with a lower level of sexual satisfaction in people coping with MS. Similar results were obtained in studies by Kołtuniuk and colleagues [13] and Marck et al. [14]. 

The results of this study indicate that individuals diagnosed with MS struggle with numerous problems, related to both physical and neurological functioning, as well as the sexual sphere. The occurring sexual dysfunctions were significantly associated with a reduced level of sexual well-being, which is an important factor influencing the overall assessment of the quality of life in a group of people with various types of disorders resulting in the occurrence of disability [23]. As mentioned before, studies indicate that over 50% of women assess that their sexual life has not changed after the diagnosis of MS [21]. The tested polynomial (non-linear) effects suggest that the studied group could be characterized by many potential buffering factors (personal resources), which would buffer the effects of MS and its symptoms on sexual well-being in the studied group. Previous studies on Polish patients with MS showed that personal resources such as resilience, sense of coherence, or acceptance of illness [6,7,34,35,36] play an important role in the quality of life and well-being of individuals diagnosed with MS and are associated with the severity of the physical and neurological disability. It is possible that those personal resources also play an important role in the relationship between disability and sexual well-being. 

This claim has a strong background in the theoretical models, such as Aaron Antonovsky’s [37,38,39] model of salutogenesis. In his opinion, health and disease constitute a continuum, on which avoiding the risk factors of a given disease is not enough to achieve full health. An important role in achieving health is played by preventive factors (salutary factors), which are responsible for promoting health, instead of reducing risk factors. According to Antonovsky [37,38,39], the level of health depends on four factors: generalized resistance resources (GRRs), stressors, lifestyle, and sense of coherence. Generalized resistance resources facilitate effective coping with the stressors of our daily lives and perceiving the world as meaningful.

Every research project has its limitations. The described study is cross-sectional and, to verify the obtained results, longitudinal studies should be conducted. The studied sample is also not representative due to sociodemographic and biomedical variables. Moreover, the subject of the study could have attracted a very special group of people interested in the sexual sphere, or only those who are active in it, which does not show the full picture of sexual well-being in multiple sclerosis. Furthermore, the used measure of sexual well-being does not take into account the individual aspects of chronic disease. Therefore, further research should focus on developing and using methods for measuring sexual well-being that will take into account the limitations resulting from chronic disease. Lastly, it is also not known what “sexual activity” was for the respondents—perhaps they also included kissing, touch, taking care of closeness and intimacy, and not sex with penetration, in which sexual dysfunctions and disabilities could interfere. This means that further studies should thoroughly explore the sphere of sexual activity of people with multiple sclerosis. Nevertheless, this study highlights the important role of the sexual sphere in the lives of people with MS.

## 5. Conclusions

Individuals suffering from MS can face many neurological and physical difficulties. They can be related to the occurrence of sexual dysfunctions, which can significantly impact one’s sexual life. Data suggest that the subject of sexual dysfunctions is often omitted during consultations with MS patients [17]. This study suggests that the occurrence of sexual dysfunctions can be an important subject in MS patients’ sexual well-being. It underlines the fact that the subject of one’s sexuality cannot be omitted while providing medical and psychological support for individuals with MS. Medical consultations on the subject of sexual dysfunctions are important for those patients [16]. The results of this study may have practical application in the area of psychoeducation and sexual education, both in the group of patients with MS and medical personnel. Our study showed that sexual dysfunctions were the most important predictor of sexual well-being in the studied group of MS patients. Psychoeducation in this area can especially help patients maintain optimal levels of sexual well-being. Knowledge of sexual dysfunctions and a way of coping with them may significantly improve the sexual satisfaction of people with MS and may lead to an improvement in their overall quality of life by drawing attention to an area of life that they may sometimes forget about. Furthermore, our study showed that the levels of neurological and physical disability do not have to be related to one’s sexual well-being. This is also highly important in the case of patients’ psychoeducation. Our results show that despite many physical and neurological difficulties, individuals can lead satisfactory sexual lives. Some patients are very focused on the impact of their disease on their lives especially those who do not accept it. Therefore, psychoeducational programs for individuals with MS should underline the fact that disability in various areas does not have to impact their well-being in many spheres. Since sexual dysfunctions were the most important predictor of sexual well-being in our study, the education of patients with MS should also be focused on how to deal with sexual problems when they occur.

## Figures and Tables

**Table 1:** Gender differences in the studied variables.

	Women		Men		*t* (111)	*p*	*d*
	M	SD	M	SD			
Age	43.08	11.39	47.82	11.72	−2.17	0.032	0.41
MS duration	11.06	6.29	12.88	7.79	−1.36	0.177	0.26
Neurological disability	17.09	7.19	17.60	8.05	−0.35	0.727	0.07
Physical disability	4.96	1.72	4.17	1.86	2.35	0.021	0.44
Sexual dysfunctions	1.72	1.85	1.08	1.77	1.86	0.066	0.35
Sexual well-being	12.87	7.20	13.70	8.18	−0.57	0.570	0.11

**Table 2:** Relationships between studied variables.

	Shapiro–Wilk’s *W*	*p*	*K*	*SKE*	1.	2.	3.	4.	5.
Age	0.97	0.005	−0.79	−0.33	-				
2. MS duration	0.95	<0.001	0.87	0.86	0.52 ***	-			
3. Neurological disability	0.98	0.077	−0.05	0.43	0.19 *	0.16	-		
4. Physical disability	0.95	<0.001	−1.16	−0.05	0.01	0.16	0.40 ***	-	
5. Sexual dysfunctions	0.74	<0.001	−0.71	0.93	0.06	0.10	0.45 ***	0.05	-
6. Sexual well-being	0.90	<0.001	−0.23	0.82	−0.12	−0.08	−0.27 **	−0.01	−0.40 ***

* *p* < 0.05; ** *p* < 0.01; *** *p* < 0.001

**Table 3:** Results of the linear and polynomial regression analyses.

**Linear regression** *F* = 11.39; *df*_1_ = 2; *df*_2_ = 110; *p* < 0.001; adj. *R*^2^ = 0.16					
**DV: Sexual well-being**	***beta***	***p***	***eta*** ^**2**^ *_**p**_*	***omega*** ^**2**^	**adj.*****R***^**2**^ (**Cohen’s*****f***^**2**^)
Neurological disability	−0.12	0.229	0.01	<0.01	0.16
Sexual dysfunctions	−0.35	<0.001	0.10	0.09	(0.19)
**Polynomial regression** with two tested non-linear effects: quadratic and cubic					
**DV: Sexual well-being**	***F***	***p***	***eta*** ^**2**^ *_**p**_*	***omega*** ^**2**^	**adj.** ***R*** ^**2**^
Age	1.63	0.200	0.03	0.01	0.01
MS duration	1.14	0.324	0.02	0.01	<0.01
Neurological disability	3.80	0.025	0.06	0.05	0.05
Physical disability	0.81	0.446	0.01	<0.01	<0.01
Sexual dysfunctions: quadratic function	1.27	0.263	0.01	<0.01	<0.01
Sexual dysfunctions: cubic function	<0.01	0.979	<0.01	<0.01	<0.01

Note: model fit coefficients for quadratic and cubic effects of the number of sexual dysfunctions are reported separately due to the significant results obtained in linear regression; otherwise, the values of the entire model would represent the results of a linear model and not a non-linear model.

## Data Availability

The datasets presented in this article are not readily available because the data are part of an ongoing study. The raw data supporting the conclusions of this article will be made available by the authors on request.

## References

[1] Wallin M.T., Culpepper W.J., Nichols E., Bhutta Z.A., Gebrehiwot T.T., Hay S.I., Khalil I.A., Krohn K.J., Liang X., Naghavi M. (2019). Global, Regional, and National Burden of Multiple Sclerosis 1990–2016: A Systematic Analysis for the Global Burden of Disease Study 2016. Lancet Neurol..

[2] Polish National Health Fund (2021). NFZ o Zdrowiu. Stwardnienie Rozsiane [National Health Fund on Health. Multiple Sclerosis].

[3] Gałązka-Sobotka M., Gierczyński J., Gryglewicz J., Rejdak K., Sławek J., Słowik A., Adamczyk-Sowa M., Bartosik-Psujek H., Wieczorek M., Kułakowska A. (2021). The Multiple Sclerosis Patient’s Journey in Poland–Current Diagnosis. Curr. Neurol..

[4] Reich D.S., Lucchinetti C.F., Calabresi P.A. (2018). Multiple Sclerosis. N. Engl. J. Med..

[5] Bass A.D., Van Wijmeersch B., Mayer L., Mäurer M., Boster A., Mandel M., Mitchell C., Sharrock K., Singer B. (2019). Effect of Multiple Sclerosis on Daily Activities, Emotional Well-Being, and Relationships: The Global vsMS Survey. Int. J. MS Care.

[6] Dymecka J., Gerymski R., Tataruch R., Bidzan M. (2022). Sense of Coherence and Health-Related Quality of Life in Patients with Multiple Sclerosis: The Role of Physical and Neurological Disability. J. Clin. Med..

[7] Dymecka J., Gerymski R. (2020). Akceptacja Choroby Jako Mediator Zależności Pomiędzy Niepełnosprawnością Neurologiczną a Jakością Życia Uwarunkowaną Stanem Zdrowia Osób Ze Stwardnieniem Rozsianym [Acceptance of Illness as a Mediator of the Relationship between Neurological Disability and Health-Related Quality of Life of People with Multiple Sclerosis]. Neuropsychiatry Neuropsychol..

[8] Furmańska J., Koziarska D., Szcześniak M., Rzepa T., Nowacki P. (2017). Sexual Satisfaction, Self-Esteem and Acceptance of Illness among Relapse-Remitting Multiple Sclerosis Patients. Adv. Psychiatry Neurol..

[9] Magyari M., Koch-Henriksen N. (2022). Quantitative Effect of Sex on Disease Activity and Disability Accumulation in Multiple Sclerosis. J. Neurol. Neurosurg. Psychiatry.

[10] Zhao Z., Zhang Y., Du Q., Chen H., Shi Z., Wang J., Qiu Y., Yan C., Zhou H. (2021). Differences in Physical, Mental, and Social Functions between Males and Females in Multiple Sclerosis: A Multicenter Cross-Sectional Study in China. Mult. Scler. Relat. Disord..

[11] Lew-Starowicz M., Rola R. (2014). Correlates of Sexual Function in Male and Female Patients with Multiple Sclerosis. J. Sex. Med..

[12] Dastoorpoor M., Zamanian M., Moradzadeh R., Nabavi S.M., Kousari R. (2021). Prevalence of Sexual Dysfunction in Men with Multiple Sclerosis: A Systematic Review and Meta-Analysis. Syst. Rev..

[13] Kołtuniuk A., Przestrzelska M., Karnas A., Rosińczuk J. (2020). The Association Between Sexual Disorders and the Quality of Life of Woman Patients With Multiple Sclerosis: Findings of a Prospective, Observational, and Cross-Sectional Survey. Sex. Med..

[14] Marck C.H., Jelinek P.L., Weiland T.J., Hocking J.S., De Livera A.M., Taylor K.L., Neate S.L., Pereira N.G., Jelinek G.A. (2016). Sexual Function in Multiple Sclerosis and Associations with Demographic, Disease and Lifestyle Characteristics: An International Cross-Sectional Study. BMC Neurol..

[15] Nazari F., Shaygannejad V., Mohammadi Sichani M., Mansourian M., Hajhashemi V. (2020). Sexual Dysfunction in Women with Multiple Sclerosis: Prevalence and Impact on Quality of Life. BMC Urol..

[16] Calabrò R.S. (2019). When Healthcare Providers Do Not Ask, Patients Rarely Tell: The Importance of Sexual Counselling in Multiple Sclerosis. J. Natl. Med. Assoc..

[17] Prévinaire J.G., Lecourt G., Soler J.M., Denys P. (2014). Sexual Disorders in Men with Multiple Sclerosis: Evaluation and Management. Ann. Phys. Rehabil. Med..

[18] Dehghan-Nayeri N., Khakbazan Z., Ghafoori F., Nabavi S.M. (2017). Sexual Dysfunction Levels in Iranian Women Suffering from Multiple Sclerosis. Mult. Scler. Relat. Disord..

[19] Ghajarzadeh M., Jalilian R., Mohammadifar M., Sahraian M.A., Azimi A. (2014). Sexual Function in Women with Multiple Sclerosis. Acta Med. Iran..

[20] Prinssen P., Jongen P.J., Heerings M., Wyverkens E., T’Sjoen G., Deschepper E., Dewitte M. (2023). Sexual Motivation in Persons with Multiple Sclerosis: A Controlled Cross-Sectional Study. Degener. Neurol. Neuromuscul. Dis..

[21] Lew-Starowicz M., Rola R. (2013). Prevalence of Sexual Dysfunctions Among Women with Multiple Sclerosis. Sex. Disabil..

[22] Mohammadi K., Rahnama P., Rafei Z., Ebrahimi-Aveh S.M., Montazeri A. (2020). Factors Associated with Intimacy and Sexuality among Young Women with Multiple Sclerosis. Reprod. Health.

[23] Gerymski R., Cisek A., Dymecka J. (2022). Seksualny Dobrostan a Satysfakcja z Życia Osób z Niepełnosprawnością Ruchową: Moderacyjna Rola Samooceny [Sexual Well-Being and Life Satisfaction of People with Motor Disabilities: The Moderating Role of Self-Esteem]. J. Sex. Ment. Health.

[24] Gerymski R., Latusek-Mierzwa M. (2024). Network Analysis of Sexual Well-Being in Women with Heart Failure: The Psychocardiological Perspective. Healthcare.

[25] Gerymski R., Szeląg M. (2023). Sexual Well-Being in Individuals with Schizophrenia: A Pilot Study on the Role of Self-Esteem and Acceptance of Illness. Eur. J. Investig. Health Psychol. Educ..

[26] Gerymski R. (2021). Short Sexual Well-Being Scale–a Cross-Sectional Validation among Transgender and Cisgender People. Health Psychol. Rep..

[27] Sharrack B., Hughes R.A. (1999). The Guy’s Neurological Disability Scale (GNDS): A New Disability Measure for Multiple Sclerosis. Mult. Scler. J..

[28] Kurtzke J.F. (1983). Rating Neurologic Impairment in Multiple Sclerosis: An Expanded Disability Status Scale (EDSS). Neurology.

[29] (2024). JAMOVI.

[30] Kister I., Bacon T., Cutter G.R. (2021). How Multiple Sclerosis Symptoms Vary by Age, Sex, and Race/Ethnicity. Neurol. Clin. Pract..

[31] Romano D., Zemon V., Foley F.W. (2023). Age-Related Differences in the Severity of Sexual Dysfunction Symptoms and Psychological Distress in Individuals with Multiple Sclerosis. Mult. Scler. Relat. Disord..

[32] Alehashemi A., Mostafavian Z., Dareini N. (2019). Sexual Function in Iranian Female Multiple Sclerosis Patients. Open Access Maced. J. Med. Sci..

[33] Kaplan T.B., Feldman T., Healey B., Behn M., Glanz B., Chitnis T. (2023). Sexual Problems in MS: Sex Differences and Their Impact on Quality of Life. Mult. Scler. Relat. Disord..

[34] Dymecka J., Gerymski R. (2020). Role of Resiliency in the Relationship between Disability and Quality of Life of People with Multiple Sclerosis: Mediation Analysis. Adv. Psychiatry Neurol..

[35] Dymecka J., Gerymski R., Bidzan M. (2020). Selected Psychometric Aspects of the Polish Version of the Liverpool Self-Efficacy Scale. Curr. Issues Personal. Psychol..

[36] Dymecka J., Bidzan M., Gerymski R. (2022). Severity of Biomedical Variables Associated with the Course of Multiple Sclerosis and Levels of Mental Resiliency. Neuropsychiatr. Neuropsychol. Neuropsychol..

[37] Antonovsky A. (1979). Health, Stress, and Coping.

[38] Antonovsky A. (1987). Unraveling the Mystery of Health: How People Manage Stress and Stay Well.

[39] Antonovsky A. (1996). The Salutogenic Model as a Theory to Guide Health Promotion. Health Promot. Int..

